# Mediating roles of weight stigma and physical activity avoidance in the associations between severity of gaming disorder and levels of physical activity among young adults

**DOI:** 10.1556/2006.2024.00083

**Published:** 2025-01-24

**Authors:** Mohsen Saffari, Chi-Hsien Huang, Po-Ching Huang, Yun-Hsuan Chang, Jung-Sheng Chen, Wai Chuen Poon, Marc N. Potenza, Mark D. Griffiths, Chung-Ying Lin

**Affiliations:** 1 Health Research Center, Life Style Institute, Baqiyatallah University of Medical Sciences, Tehran, Iran; 2 Health Education Department, Faculty of Health, Baqiyatallah University of Medical Sciences, Tehran, Iran; 3 Department of Family Medicine and Community Medicine, E-Da Hospital, I-Shou University, Kaohsiung, Taiwan; 4 School of Medicine, College of Medicine, I-Shou University, Kaohsiung, Taiwan; 5 College of Nursing, Kaohsiung Medical University, Kaohsiung, Taiwan; 6 School of Physical Therapy, Graduate Institute of Rehabilitation Science, College of Medicine, Chang Gung University, Taoyuan, Taiwan; 7 Department of Physiotherapy, School of Nursing and Health Studies, Hong Kong Metropolitan University, Hong Kong; 8 Institute of Gerontology, College of Medicine, National Cheng Kung University, Tainan, Taiwan; 9 Institute of Behavioral Medicine, College of Medicine, National Cheng Kung University, Tainan, Taiwan; 10 Department of Psychology, National Cheng Kung University, Tainan, Taiwan; 11 Department of Psychiatry, National Cheng Kung University Hospital, Yunlin, Taiwan; 12 Institute of Genomics and Bioinformatics, National Chung Hsing University, Taichung, Taiwan; 13 Department of Medical Research, E-Da Hospital, I-Shou University, Kaohsiung, Taiwan; 14 Sunway Business School, Sunway University, Selangor Darul Ehsan, Malaysia; 15 Department of Psychiatry, School of Medicine, Yale University, New Haven, CT, USA; 16 Connecticut Mental Health Center, New Haven, CT, USA; 17 Connecticut Council on Problem Gambling, Wethersfield, CT, USA; 18 Child Study Center, Yale School of Medicine, New Haven, CT, USA; 19 Department of Neuroscience, Yale University, New Haven, CT, USA; 20 Wu Tsai Institute, Yale University, New Haven, CT, USA; 21 International Gaming Research Unit, Psychology Department, Nottingham Trent University, Nottingham, UK; 22 Institute of Allied Health Sciences, College of Medicine, National Cheng Kung University, Tainan, Taiwan; 23 Biostatistics Consulting Center, National Cheng Kung University Hospital, College of Medicine, National Cheng Kung University, Tainan, Taiwan; 24 Department of Public Health, College of Medicine, National Cheng Kung University, Tainan, Taiwan; 25 Department of Occupational Therapy, College of Medicine, National Cheng Kung University, Tainan, Taiwan

**Keywords:** addictive behaviors, videogames, gaming disorder, physical activity, weight stigma, youth

## Abstract

**Background and aims:**

There are limited data regarding associations between gaming disorder and physical activity (PA). The present study investigated the direct association between these two variables and assessed the potentially mediating roles of PA avoidance and two types of weight stigma (i.e., internalized weight stigma and perceived weight stigma) in the association.

**Methods:**

An online cross-sectional survey that assessed PA avoidance, two types of weight stigma, and PA level was completed in late 2023 by 884 Taiwanese young adults aged between 20 and 40 years (63.9% females). Multinomial logistic regression and structural equation modeling (SEM) were used to assess the associations between variables and perform the mediation analysis.

**Results:**

Cognitive behavioral symptoms and negative consequences related to gaming disorder were more common among participants with lower PA than those with moderate to high PA. Individuals at risk of gaming disorder exhibited higher level of PA avoidance, internalized weight stigma, and perceived weight stigma. The SEM found a direct association between gaming disorder and PA, which was negatively mediated by PA avoidance. However, this direct effect was not present when the association was negatively mediated by serial mediations of weight stigma and PA avoidance.

**Discussion and Conclusions:**

Higher gaming disorder was associated with higher levels of PA, but this association may not be present when taking into account the mediation effect of weight stigma and PA avoidance. The findings suggest complex relationships and further research is needed to examine individual differences and relationships among clinical groups.

## Introduction

Although playing videogames provides enjoyment and can be beneficial to many individuals, spending a long time playing such games may negatively impact both mental and physical health (Meng et al., 2022). Gaming disorder (GD) was officially included in the 11th edition of the *International Classification of Diseases* (ICD-11) in 2019 (World Health Organization, 2019). GD is characterized by persistent or increasing priority given to gaming leading to impaired control and continued/escalating gaming despite adverse consequences (Wang, Ren, Long, Liu, & Liu, 2019). Criteria for a related condition (internet gaming disorder [IGD]) were defined in the fifth edition of the *Diagnostic and Statistical Manual of Mental Disorders* (DSM-5) (American Psychiatric Association, 2022). Individuals with GD prioritize gaming over other important life activities such as eating, sleeping, caring about personal hygiene, and exercise/physical activity (PA) (Bäcklund, Elbe, Gavelin, Sörman, & Ljungberg, 2022; Stevens, Dorstyn, Delfabbro, & King, 2021). They may also engage in fewer real-world social interactions and experience problems in performing their familial, academic, or occupational responsibilities (Huot-Lavoie et al., 2023). Irritability, depression, anxiety, and boredom also are common among individuals with GD (Bäcklund et al., 2022; Gavriel-Fried et al., 2023). A meta-analysis of 53 studies comprising over 226,000 participants reported the prevalence of GD to be 3.05%. However, when using only those studies with the highest quality of data, the prevalence was 1.96% (Mihara et al., 2017; Stevens et al., 2021).

Studies have shown that although all age groups engage in online gaming for entertainment in their everyday lives (Stevens et al., 2021), videogame playing is more prevalent among those in earlier developmental stages from preschool periods to young adulthood (André, Broman, Håkansson, & Claesdotter-Knutsson, 2020). Moreover, younger people appear to be more susceptible to excessive gaming and gaming disorder. In a recent meta-analysis of more than 150 studies worldwide, Gao, Wang, and Dong (2022) reported a pooled prevalence of 10% for IGD among adolescents and young adults. A further subgroup analysis indicated a higher prevalence of IGD among young adults compared to adolescents. However, in another recent meta-analysis that assessed all types of GD in different age groups, the overall pooled prevalence of GD was approximately 3% (Kim et al., 2022), suggesting that in comparison to previous studies, some kinds of GD (such as IGD) may be more prevalent among young adults.

Playing videogames, particularly non-active ones, typically involves low energy consumption and may reduce PA. Therefore, playing videogames may lead to increased body fat and promote sedentary lifestyles that may increase the likelihood of health problems including musculoskeletal disorders, diabetes, and cardiovascular concerns (Robinson, Daly, Ridgers, & Salmon, 2015). Consequently, GD may result in individuals engaging in physically inactive lifestyles that may contribute to serious psychological and physical health challenges (Alanko, 2023; Jorgenson, Hsiao, & Yen, 2016). However, contrary to the negative association between GD and PA (Cheng, Wang, Wang, Zhang, & Qin, 2022; Kwok, Leung, Poon, & Fung, 2021; Pirnes et al., 2022), some studies have reported a positive association between GD and PA (Alpe et al., 2024; Huang et al., 2022), suggesting this association may be influenced by other underlying factors.

PA often exerts positive effects on both mental health (e.g., increased motivation and vitality and reduction of anxiety/depression) and physical health (Warburton et al., 2017). As individuals with GD tend to show lower engagement in PA (Bäcklund et al., 2022; Wang et al., 2019), understanding the potential mechanisms or pathways that may mediate the association between GD and PA could help identify strategies to prevent the negative impacts of gaming on health and improve PA among those affected by GD. Arguably, research to date has mostly focused on socioeconomic and environmental factors as primary barriers to adequate PA (Hesketh, Lakshman, & van Sluijs, 2017). However, other factors, including psychological motives involving self-concepts and concerns related to physical appearance may also influence decisions to avoid PA and exercise (Bevan et al., 2021; Palenzuela-Luis, Duarte-Clíments, Gómez-Salgado, Rodríguez-Gómez, & Sánchez-Gómez, 2022). When individuals are less satisfied with their body shape and think others may mock or shame them due to their poor physical fitness (including being overweight/obese with related weight stigma), they may either decide to engage in PA to address the concerns or avoid PA due to feeling overwhelmed. PA avoidance can be viewed as a psychological construct regarding individuals not wanting to participate in any PA due to appearance-related issues (Hartmann et al., 2010; More et al., 2019). This was formally investigated by Bevan et al. (2022) who developed a specific instrument to assess PA avoidance. Moreover, recent research using this instrument has found that PA avoidance is associated with both weight stigma and GD (Fan et al., 2023; Lin et al., 2024; Saffari et al., 2023). Therefore, considering PA, PA avoidance, and weight stigma in relation to GD is important (Ajibewa et al., 2022; Bevan et al., 2021).

Weight stigmatization may occur in different settings including schools, workplaces, healthcare centers, mass media, and interpersonal communications. Negative attitudes or beliefs regarding a person's weight may promote rejection, prejudice, and discrimination (Bevan et al., 2021). Negative comments to individuals from others regarding their weight, physical barriers (e.g., being unfit to use exercise devices), being excluded by peers, or being ignored during PA, are common stigmatizing events that may prevent individuals from participating in PA (Lucibello, Sabiston, Pila, & Arbour-Nicitopoulos, 2023). Weight stigma has been positively associated with psychological distress and eating disorders, and negatively associated with motivation to engage in PA, leading to PA avoidance (Huang et al., 2024; Westby, Jones, & Loth, 2021). Unpleasant memories of experiencing stigmatization may reduce desires to enter situations or settings such as exercise clubs or gyms that may put previously stigmatized individuals at elevated risk of further discrimination (Puhl, Himmelstein, & Pearl, 2020). Therefore, weight stigma may increase the risk of gaining weight, remaining overweight/obese, and experiencing related conditions such as type II diabetes and cardiovascular diseases (Westby et al., 2021).

Weight stigma may interfere with maintaining a normal weight, especially among individuals with obesity (Schafer et al., 2011). Weight stigma has been associated with greater caloric intake, non-adherence to diets, and less-than-recommended levels of moderate to vigorous PA (Meadows et al., 2020; Puhl et al., 2020). Therefore, both perceived weight stigma and internalized weight stigma may influence PA. Although both positive and negative relationships may exist between weight stigma and PA, internalized weight stigma, which occurs when individuals accept or believe negative social beliefs regarding their weight status (Meadows et al., 2020), has been more frequently associated with lower levels of PA (Pearl et al., 2015, 2020, 2021; Puhl, Quinn, Weisz, & Suh, 2017). However, no previous studies have investigated how weight stigma may mediate associations between GD and PA.

Although there are only a few studies that have examined the associations between GD and weight stigma, the current evidence supports the contention that GD may be a contributor to weight gain and the development of weight stigma. Kamolthip et al. (2023) found university students' weight status was positively associated with perceived weight stigma and GD, and also reported a significant association between perceived weight stigma and excessive gaming. In another study, Chen, Chen, O'Brien, Latner, and Lin (2021) assessed psychological distress and online behaviors among schoolchildren who were overweight and not overweight. They also found that children who were overweight had higher levels of weight stigma, and that weight stigma was greater among those with higher rates of problematic internet use behaviors (Chen et al., 2021). Moreover, there are several studies which have reported that problematic gaming behavior may be associated with weight status of adolescents and young people, which suggest there is a relationship between GD and weight stigmatization (Phan et al., 2019; Simons et al., 2015; Stubblefield et al., 2017).

Given that associations between GD and PA have been established in several studies, mediation analysis would help explore underlying mechanisms or stages by which an independent factor like GD may influence PA through intermediate variables (e.g., weight stigma and PA avoidance motivations). Indeed, indirect effects between GD and PA through intermediate variables may provide a clearer understanding of the nature of such relationships and provide potential targets for interventions. Considering the contemporary aesthetic of ‘thin beauty’ that is now prevalent in Eastern countries (Cheng et al., 2018; Huang et al., 2024) and studies suggesting that young people with GD may be less likely to engage in PA due to psychological factors such as weight stigmatization, the present study investigated the direct and indirect effects of GD on PA and examined how weight stigma and PA avoidance may mediate the relationship between GD and PA.

**Fig. 1.**
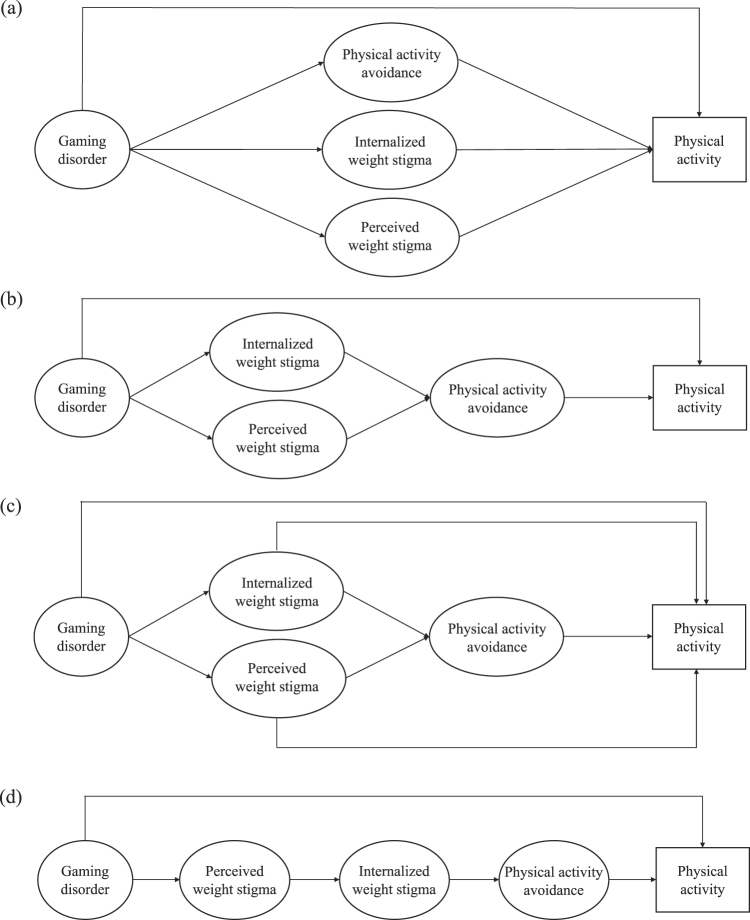
Framework of proposed models: (a) the parallel mediation model and (b, c, d) the serial mediation model

It was hypothesized that in addition to (i) the direct association between GD and PA (H_1_), there would be indirect associations between GD and PA through the parallel mediation of (ii) PA avoidance (H_2_), (iii) internalized weight stigma (H_3_), and (iv) perceived weight stigma (H_4_) (Fig. 1a); the serial mediation of (v) internalized weight stigma then PA avoidance (H_5_) and (vi) perceived weight stigma then PA avoidance (H_6_) (Fig. 1b and c); and the serial mediation of (vii) perceived weight stigma, internalized weight stigma, then PA avoidance (H_7_) (Fig. 1d).

## Methods

### Participants and procedure

A cross-sectional online survey was conducted using convenience and snowball sampling. Data were collected via the *SurveyMonkey* platform between September and December 2023. Participants aged between 20 and 40 years were eligible to participate. The authors distributed the online survey via university faculties in Taiwan. Participants were encouraged to distribute the survey link to whoever met the eligibility criteria. The study was conducted in accordance with the Declaration of Helsinki. Prior to starting the survey, informed consent was provided. By clicking ‘*Yes*’, individuals gave their informed consent and could participate in the study. The second page of the survey asked if the individual was aged between 20 and 40 years. If they clicked ‘*No*’, the survey ended immediately. A total of 887 participants were initially recruited. Three participants who preferred not to disclose their sex were excluded. Therefore, a total of 884 participants (565 females; 63.91%) were included in the analysis. The mean age was 28.82 years (SD = 6.06), and the average BMI was 22.82 kg/m^2^ (SD = 4.00).

### Measures

#### Demographic information

Demographic questions included (i) age (in years), (ii) sex (male or female), (iii) height (in cm), (iv) weight (in kg), (v) time spent on smartphone (hours per day), and (vi) time spent engaged in outdoor activities (hours per day). The height/weight information was then used to calculate body mass indexes (BMIs) in kg/m^2^.

#### International Physical Activity Questionnaire-Short Form (IPAQ-SF)

Weekly PA level was assessed using the IPAQ-SF (Liou, Jwo, Yao, Chiang, & Huang, 2008). Seven items assess the time spent engaging in different levels of activities (i.e., resting/sitting, walking, and moderate and vigorous levels of PA). PA levels were defined by metabolic equivalents (METs) (3.3 METs for walking, 4 for moderate PA, and 8 for high PA). Total PA time was calculated by time spent engaging at each PA level multiplied by their metabolic equivalents (METs) and summing the quantity. Individuals with PA levels higher than 3,000 MET-minutes/week were identified as exhibiting a ‘high’ levels of PA. Those with PA levels of 600 MET-minutes/week were identified as exhibiting a ‘moderate’ level of PA. Others were defined as having ‘low’ levels of PA (IPAQ Research Committee, 2015). The Chinese IPAQ-SF has demonstrated strong psychometric properties (Cheng et al., 2019; Fung et al., 2019; Saffari et al., 2022). A sample item is “*During the last 7 days, how much time did you spend sitting on a week day*?”

#### Gaming Disorder Scale for Adolescents (GADIS-A)

The GADIS-A was originally developed to assess GD among adolescents (Paschke, Austermann, & Thomasius, 2020) and has been validated for use in young adults (Wu et al., 2023). The GADIS-A includes nine items plus one time-related question, and scores on the GADIS-A are calculated for three GD domains: (F1) negative consequences, (F2) cognitive behavioral symptoms, and (F3) frequency of problematic gaming (Item 10). Nine items are rated using five-point Likert-like scales (0 = strongly disagree, 4 = strongly agree) and summed to generate total scores. Cutoffs of 5 for negative consequences, and 9 for cognitive behavioral symptoms were used to identify the problematic domains. The Chinese GADIS-A has demonstrated strong psychometric properties (Wu et al., 2023). A sample item is “*I often continue gaming even though it causes me stress with others (e.g., my parents, siblings, friends, partner, teachers)*”. The internal consistency of the GADIS-A in the present study was excellent (Cronbach's [α] = 0.953).

#### Tendency to Avoid Physical Activity Scale (TAPAS)

Tendency to avoid engaging in PA was assessed using the TAPAS (Bevan et al., 2022). Ten items rated on five-point Likert-like scales (1 = strongly disagree; 5 = strongly agree) are summed to generate total scores ranging between 5 and 50. Higher scores indicate a greater tendency to avoid participating in PA. The Chinese TAPAS has demonstrated good psychometric properties (Fan et al., 2023; Saffari et al., 2023). A sample item is “*I worry about participating in sports because I don't like how my body looks when playing sports*”. The internal consistency of the TAPAS in the present study was excellent (*α* = 0.948).

#### Weight Bias Internalization Scale (WBIS)

The level of internalized weight stigma was assessed using the WBIS (Durso et al., 2012). For the Chinese version, all items were modified by changing the concept of ‘overweight’ into ‘weight’ as suggested by the developer. Eleven items are rated on five-point Likert-like scales (1 = completely disagree; 5 = completely agree) and summed to generate a total score ranging between 11 and 55. Higher scores reflect more weight-related discrimination towards oneself. The Chinese WBIS has demonstrated good psychometric properties (Lin et al., 2019; Pakpour et al., 2019). A sample item is “*I wish I could drastically change my weight*”. The internal consistency of the WBIS in the present study was excellent (*α* = 0.906).

#### Perceived Weight Stigma Scale (PWSS)

The degree of weight stigma perceived by individuals was assessed using the PWSS (Schafer et al., 2011). Ten items are rated dichotomously (0 = no; 1 = yes) and summed to generate a total score ranging between 0 and 10. Higher scores reflect greater weight stigma. The Chinese PWSS has demonstrated adequate psychometric properties (Lin et al., 2020). A sample item is “*Because of your weight, people act as if you are inferior*”. The internal consistency of the PWSS in the present study was very good (*α* = 0.866).

### Data analysis

Participants were divided into three groups based on their PA level based on IPAQ-SF classification (i.e., low, moderate, and high PA). The GADIS-A domain scores were further used to examine if a participant was at risk of GD regarding negative consequences (GADIS-A F1) or cognitive behavioral symptoms (GADIS-A F2). Baseline characteristics were described using descriptive statistics. Analyses of variance (ANOVAs) with Bonferroni correction and chi-square tests were used to compare the study variables between groups. Correlations between variables were calculated using Pearson correlation coefficients. Relationships between levels of PA and other study variables were calculated using multinomial logistic regression. The low-PA group was used as the reference group to compare the study variables with regard to moderate and high levels of PA. In the multinomial logistic regression model, age, sex (reference group: male), BMI, and GADIS-A F1 (reference group: no/low risk), GADIS-A F2 (reference group: low risk), TAPAS, WBIS, and PWSS scores were entered as independent variables. Multiple linear regression was conducted using TAPAS scores as a dependent variable and age, gender (reference group: male), BMI, GADIS-A F1 (reference group: low risk), GADIS-A F2 (reference group: low risk), WBIS, and PWSS scores as independent variables. Moreover, GADIS-A F1 and GADIS-A F2 were used for both multinominal and multiple linear regressions because this approach provided detailed information regarding how different GD domains were associated with PA.

Moderation and mediation models were then analyzed (and in both the moderation and mediation models, the GADIS-A was used to assess the construct of GD as a whole to understand how GD was associated with PA). In the moderation models, the moderated effects of PA avoidance, internalized weight stigma, perceived weight stigma in the association between GD and PA scores were examined using Hayes' Process Macro (Model 1) (Hayes, 2013). Each variable was added as a moderator in the separate models, while the other two were controlled for as covariates (for example, when testing the moderated effects of PA avoidance, internalized weight stigma and perceived weight stigma were controlled for in the model).

Regarding the mediation model, four models were analyzed: the first model proposed a parallel mediation model with parallel mediated effects of PA avoidance, internalized weight stigma, and perceived weight stigma (Fig. 1a); the second model proposed the first serial mediation model having serial mediated effects from two types of weight stigma to PA avoidance without direct effects from GD to PA (Fig. 1b); the third model proposed the second serial mediation model having serial mediated effects from two types of weight stigma to PA avoidance with direct effects from GD to PA (Fig. 1c); and the fourth model proposed the third serial mediation model having serial mediated effects from perceived weight stigma, internalized weight stigma, to PA avoidance (Fig. 1d).

All proposed models with indirect effects of TAPAS, WBIS, and PWSS scores in the association between GADIS-A and IPAQ-SF were examined using structural equation modeling (SEM) with the Diagonally Weighted Least Squares (DWLS) estimator. Age and BMI were included as covariates. Moreover, for all models, item scores from the GADIS-A, WBIS, PWSS, and TAPAS were used as observed variables to construct the latent constructs of GD, internalized weight stigma, perceived weight stigma, and PA avoidance, respectively. The three categories in the IPAQ-SF (i.e., low, moderate and high PA) were used as observed variables in all models. Four indices tested the supportiveness: comparative fit index (CFI) and Tucker-Lewis index (TLI) should be higher than 0.9; root mean square error of approximation (RMSEA) and standardized root mean squared residual (SRMR) should be lower than 0.07 and 0.08, respectively (Hooper, Coughlan, & Mullen, 2008). After ensuring any or both of the proposed models were supported, the mediating effects of the TAPAS, WBIS and PWSS were examined using 599 bootstrapping resamples (Wilcox, 2010). When bootstrapping 95% confidence intervals (CI) did not include 0, indirect effects were considered significant (Ramachandran et al., 2020). *lavaan* for R software was used for conducting the SEM, and SPSS 29.0 was used for performing other statistical analyses. Except for *p*-values using Bonferroni correction having been adjusted to 0.017, other *p*-value significance was set at 0.05.

### Ethics

Participation in the study was voluntary, and informed consent was obtained. All answers and personal information were treated confidentially. The study was approved by the National Cheng Kung University Human Research Ethics Committee (Approval number: NCKU HREC-E-111-563-2).

## Results

Baseline characteristics and between-group comparisons are summarized in Tables 1 and 2. After dividing into three groups according to the PA level, female participants remained the majority among the three groups (ranging between 62.89% and 67.97%). The low-PA group spent the most time on their smartphone per day (5.83 h, SD = 3.76) when compared to moderate- and high-PA groups (*F* = 5.73, *p* = 0.004). The high-PA group spent the most of time engaging in outdoor activities (2.48 h, SD = 2.46) when compared to low- and moderate-PA groups (*F* = 36.27, *p* < 0.001). Negative consequences of gaming were found in 31 participants in the low-PA group (13.90%), 22 participants in the moderate-PA group (7.83%), and 24 participants in the high-PA group (6.32%) (*p* = 0.005). Gaming-related cognitive behavioral concerns were found in 73 participants in the low-PA group (32.74%), 56 participants in the moderate-PA group (19.93%), and 95 participants in the high-PA group (25.00%) (*p* = 0.004).

**Table 1.** Baseline characteristics of participants (*N* = 884)

	Total (*N* = 884)	Low (*N* = 223)	Moderate (*N* = 281)	High (*N* = 380)	*p-*value	Post hoc
Age (in years)	28.82 (6.06)	30.31 (5.98)	28.10 (6.12)	28.50 (5.91)	<0.001	Low > Moderate
						Low > High
Gender (male, %)	319 (36.0)	80 (35.87)	98 (34.88)	141 (37.11)	0.838	–
BMI	22.82 (4.00)	23.25 (4.37)	22.72 (3.78)	22.65 (3.93)	0.174	–
Smartphone time (hour per day)	5.25 (3.00)	5.83 (3.76)	5.02 (2.65)	5.09 (2.71)	0.004	Low > Moderate
						Low > High
Outdoor activity time (hour per day)	1.80 (2.20)	1.05 (1.65)	1.47 (1.95)	2.48 (2.46)	<0.001	Low < High
						Moderate < High
TAPAS (range: 5–25)	25.28 (9.71)	28.37 (10.14)	24.79 (9.45)	23.89 (9.27)	<0.001	Low > Moderate
						Low > High
WBIS (range: 11–77)	28.36 (8.42)	30.47 (8.68)	27.57 (8.06)	27.75 (8.35)	<0.001	Low > Moderate
						Low > High
PWSS (range: 0–10)	1.33 (2.27)	1.75 (2.64)	1.12 (2.00)	1.22 (2.18)	0.004	Low > Moderate
						Low > High
IPAQ-SF (MET*minutes)	3131.20 (2941.33)	503.99 (557.29)	1715.54 (779.09)	5699.54 (2706.17)	<0.001	Low < High
						Moderate < High

Data are presented as means (SDs) or frequencies (%). BMI = body mass index; TAPAS = Tendency to Avoid Physical Activity Scale; WBIS = Weight Bias Internalization Scale; PWSS = Perceived Weight Stigma Scale; IPAQ-SF = International Physical Activity Questionnaire – short form; MET = metabolic equivalent.

**Table 2.** Comparison of GADIS-A scores among three groups

	Low (*N* = 223)	Moderate (*N* = 281)	High (*N* = 380)	*p-*value
**GADIS-A F1**				0.005
Low risk	192 (86.10)	259 (92.17)	356 (93.68)	
High risk	31 (13.90)	22 (7.83)	24 (6.32)	
**GADIS-A F2**				0.004
Low risk	150 (67.26)	225 (80.07)	285 (75.00)	
High risk	73 (32.74)	56 (19.93)	95 (25.00)	

Data are presented as frequencies (%). GADIS-A F1 = Gaming Disorder Scale for Adolescents (Negative consequences domain); GADIS-A F2 = Gaming Disorder Scale for Adolescents (Cognitive behavioral symptoms domain).

Correlations and relationships between study variables and groups are shown in Tables 3 and 4. All the study variables were significantly associated with each other (*β* = −0.098 to 0.673, all *p* < 0.05), except the association between age and GADIS-A (*β* = 0.045, *p* = 0.185) and TAPAS (*β* = 0.019, *p* = 0.573), and the associations between IPAQ-SF and age (*β* = −0.026, *p* = 0.434), BMI (*β* = −0.032, *p* = 0.338), GADIS-A (*β* = 0.031, *p* = 0.360), WBIS (*β* = −0.028, *p* = 0.413) and PWSS (*β* = −0.062, *p* = 0.067). Compared to the low-PA group, the moderate-PA group was younger (odds ratio [OR] [95% CI] = 0.94 [0.92–0.97], *p* < 0.001), as was the high-PA group (OR [95% CI] = 0.95 [0.92–0.98], *p* < 0.001). The high-PA group additionally showed significantly lower odds of gaming-related consequences (OR [95% CI] = 0.48 [0.24–0.94], *p* = 0.032) and lower odds of PA avoidance (OR [95% CI] = 0.95 [0.93–0.98], *p* < 0.001).

**Table 3.** Correlation between study variables (*N* = 884)

	1	2	3	4	5	6	7
^1^ Age	–						
^2^ BMI	**0.136 (<0.001)**	–					
^3^ GADIS-A	0.045 (0.185)	**0.103 (0.002)**	–				
^4^ TAPAS	0.019 (0.573)	**0.236 (<0.001)**	**0.304 (<0.001)**	–			
^5^ WBIS	**0.074 (0.027)**	**0.420 (<0.001)**	**0.346 (<0.001)**	**0.673 (<0.001)**	–		
^6^ PWSS	**0.092 (0.006)**	**0.292 (<0.001)**	**0.298 (<0.001)**	**0.377 (<0.001)**	**0.440 (<0.001)**	–	
^7^ IPAQ-SF	−0.026 (0.434)	−0.032 (0.338)	0.031 (0.360)	**−0.098 (0.003)**	−0.028 (0.413)	−0.062 (0.067)	–

BMI = body mass index; GADIS-A = Gaming Disorder Scale for Adolescents; TAPAS = Tendency to Avoid Physical Activity Scale; WBIS = Weight Bias Internalization Scale; PWSS = Perceived Weight Stigma Scale; IPAQ-SF = International Physical Activity Questionnaire – short form. Significant findings are shown in **bold**.

**Table 4.** Multinominal logistic regression comparing study variables among three groups

Group (Ref: Low) Variable	OR (95%CI)	*p-*value
**Moderate**		
Age	**0.94 (0.92–0.97)**	**<0.001**
Gender (Ref: male)	1.03 (0.69–1.55)	0.883
BMI	1.01 (0.96–1.07)	0.597
GADIS-A F1 (Ref: low risk)	0.87 (0.42–1.77)	0.693
GADIS-A F2 (Ref: low risk)	0.79 (0.47–1.31)	0.360
TAPAS	0.98 (0.95–1.00)	0.072
WBIS	0.99 (0.95–1.02)	0.365
PWSS	0.97 (0.88–1.06)	0.449
**High**		
Age	**0.95 (0.92–0.98)**	**<0.001**
Gender (Ref: male)	0.97 (0.66–1.42)	0.880
BMI	1.00 (0.95–1.05)	0.878
GADIS-A F1 (Ref: low risk)	**0.48 (0.24–0.94)**	**0.032**
GADIS-A F2 (Ref: low risk)	1.29 (0.82–2.04)	0.276
TAPAS	**0.95 (0.93–0.98)**	**<0.001**
WBIS	1.00 (0.97–1.03)	0.954
PWSS	1.00 (0.92–1.09)	0.933

BMI = body mass index; GADIS-A F1 = Gaming Disorder Scale for Adolescents (Negative consequences domain); GADIS-A F2 = Gaming Disorder Scale for Adolescents (Cognitive behavioral symptoms domain); TAPAS = Tendency to Avoid Physical Activity Scale; WBIS = Weight Bias Internalization Scale; PWSS = Perceived Weight Stigma Scale. OR = odds ratio; Significant findings are shown in **bold**.

Results of associations between TAPAS, WBIS, and PWSS scores (i.e., the linear regression model results) are shown in Table 5. A higher level of PA avoidance was found among females (*β* = 0.06, *p* = 0.015), individuals with gaming-related cognitive behavioral concerns (*β* = 0.12, *p* < 0.001), higher internalized weight-based stigma (*β* = 0.61, *p* < 0.001), and higher perceived weight stigma (*β* = 0.08, *p* = 0.003) scores. More internalized weight-based stigma was found among females (*β* = 0.21, *p* < 0.001), individuals with lower BMIs (*β* = 0.36, *p* < 0.001), more gaming-related cognitive behavioral concerns (*β* = 0.23, *p* < 0.001), and higher perceived weight stigma (*β* = 0.28, *p* < 0.001). A higher level of perceived weight stigma was found among females (*β* = 0.15, *p* < 0.001), individuals with lower BMIs (*β* = 0.30, *p* < 0.001), more negative consequences of gaming (*β* = 0.20, *p* < 0.001), and more gaming-related cognitive behavioral concerns (*β* = 0.12, *p* < 0.001). Moreover, PA avoidance and both forms of weight stigma showed no moderating effect on the association between GD and PA (Appendix Table A1).

**Table 5.** Linear regression predicting the score of TAPAS, WBIS and PWSS

**Dependent variable: TAPAS**	***B* (SE)/*β* (*p*)**
Age	−0.06 (0.04)/−0.04 (0.105)
Gender (Ref: male)	**1.29 (0.53)/0.06 (0.015)**
BMI	−0.09 (0.07)/−0.04 (0.202)
GADIS-A F1 (Ref: low risk)	1.58 (0.98)/0.05 (0.107)
GADIS-A F2 (Ref: low risk)	**2.63 (0.66)/0.12 (<0.001)**
WBIS	**0.70 (0.03)/0.61 (<0.001)**
PWSS	**0.36 (0.12)/0.08 (0.003)**
**Fit statistics**	
F (*p*)	117.75 (<0.001)
*R*^2^ (adj. *R*^2^)	0.485 (0.481)
**Dependent variable: WBIS**	***B* (SE)/*β* (*p*)**
Age	−0.01 (0.04)/−0.004 (0.889)
Gender (Ref: male)	**3.63 (0.50)/0.21 (<0.001)**
BMI	**0.76 (0.06)/0.36 (<0.001)**
GADIS-A F1 (Ref: low risk)	−0.82 (0.95)/−0.03 (0.387)
GADIS-A F2 (Ref: low risk)	**4.38 (0.62)/0.23 (<0.001)**
PWSS	**1.03 (0.11)/0.28 (<0.001)**
**Fit statistics**	
F (*p*)	81.18 (<0.001)
*R*^2^ (adj. *R*^2^)	0.357 (0.353)
**Dependent variable: PWSS**	***B* (SE)/*β* (*p*)**
Age	0.02 (0.01)/0.05 (0.112)
Gender (Ref: male)	**0.70 (0.15)/0.15 (<0.001)**
BMI	**0.17 (0.02)/0.30 (<0.001)**
GADIS-A F1 (Ref: low risk)	**1.61 (0.28)/0.20 (<0.001)**
GADIS-A F2 (Ref: low risk)	**0.64 (0.19)/0.12 (<0.001)**
**Fit statistics**	
F (*p*)	38.95 (<0.001)
*R*^2^ (adj. *R*^2^)	0.182 (0.177)

TAPAS = Tendency to Avoid Physical Activity Scale; WBIS = Weight Bias Internalization Scale; PWSS = Perceived Weight Stigma Scale; BMI = body mass index; GADIS-A F1 = Gaming Disorder Scale for Adolescents (Negative consequences domain); GADIS-A F2 = Gaming Disorder Scale for Adolescents (Cognitive behavioral symptoms domain). Significant findings are shown in **bold**.

**Fig. 2.**
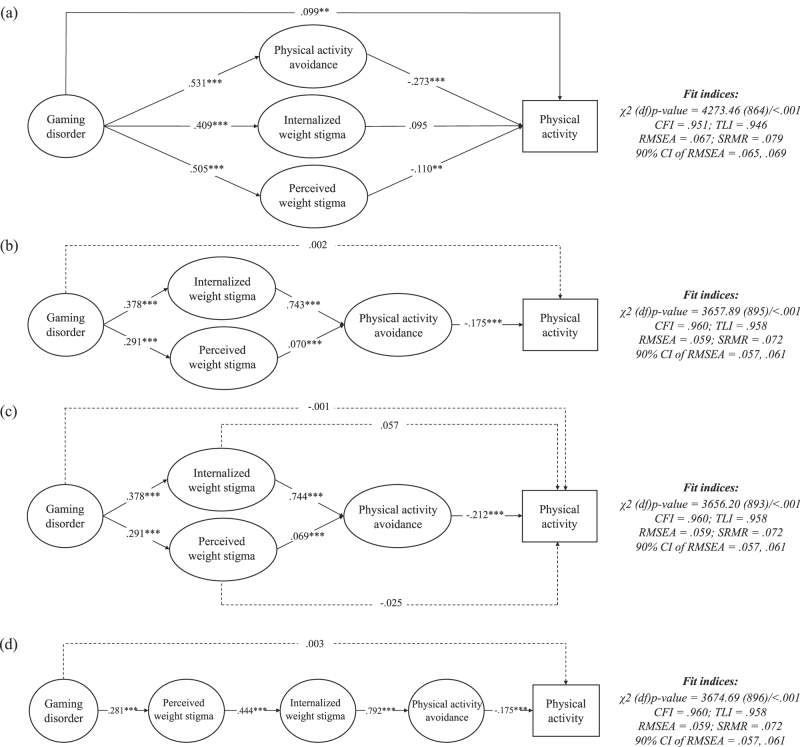
Results of structural equation modeling in examining the four proposed models. CFI = comparative fit index; TLI = Tucker-Lewis index; RMSEA = root mean square error of approximation; SRMR = standardized root mean squared residual. Data are presented using a standardized coefficient. **p < 0.01; ***p < 0.001. Solid lines indicate significance and dashed lines indicate non-significance

SEM and mediation results are shown in Fig. 2 and Table 6. All four models were acceptable based on the fit indices (CFI = 0.951 to 0.960, TLI = 0.946 to 0.958, RMSEA = 0.059 to 0.067, SRMR = 0.072 to 0.079, *χ*^*2*^ = 3656.20 to 4273.46, *χ*^*2*^/df = 4.088–4.946), except for a significant *χ*^2^ test for all models (*p* < 0.001). In the parallel mediation model (Fig. 2a), significant associations were observed between GD and PA (*β* = 0.099, *p* < 0.001), as well as PA avoidance (*β* = 0.531, *p* < 0.001), internalized weight stigma (*β* = 0.409, *p* < 0.001) and perceived weight stigma (*β* = 0.505, *p* < 0.001), which supported H_1_. In addition, both PA avoidance and perceived weight stigma were associated negatively with PA (*β* = −0.273, *p* < 0.001 for PA avoidance, and *β* = −0.110, *p* < 0.01 for perceived weight stigma). The mediation effect showed significant indirect effects of PA avoidance in the association between GD and PA (*β* = −0.145, bootstrapping 95% CI = −0.255, −0.061). These results supported H_2_, but not H_3_ or H_4_. In other words, higher GD was positively associated with PA levels, but may be negatively associated with PA level though the mediation of PA avoidance. In the first and the second serial mediation models (Fig. 2b) and (Fig. 2c), significant associations were observed in all associations (*β* = −0.212 to 0.744, all *p* < 0.001), except the direct associations between GD and PA (*β* = 0.002, *p* = 0.880 in model b and *β* = −0.001, *p* = 0.956 in model c), and the direct association between PA and (i) internalized weight stigma (*β* = 0.057, *p* = 0.267), and (ii) perceived weight stigma (*β* = −0.025, *p* = 0.316). In addition, only internalized weight stigma with PA avoidance significantly mediated the association between GD and PA (*β* = −0.046, bootstrapping 95% CI = −0.073, −0.027), which supported H_5_ but not H_6_. In the third serial mediation model (Fig. 2d), significant associations were observed in all associations (*β* = −0.175 to 0.792, all *p* < 0.001), except the direct association between GD and PA (*β* = 0.003, *p* = 0.869). Additionally, a sequential mediation effect from perceived weight stigma, internalized weight stigma, to PA avoidance was found in the association between GD and PA (*β* = −0.017, bootstrapping 95% CI = −0.028, −0.008). Therefore, H_7_ was supported.

**Table 6.** Mediation effect of PA avoidance and weight-based stigma

Mediating association	Est. (S.E.)	Std. coeff.	Bootstrapping 95% CI
GD → PA avoidance → PA	−0.183 (0.065)	**−0.145**	−0.255, −0.061
GD → IWS → PA	0.049 (0.041)	0.039	−0.019, 0.105
GD → PWS → PA	−0.070 (0.049)	−0.056	−0.146, 0.010
GD → IWS → PA avoidance → PA	−0.048 (0.011)	**−0.049**	−0.073, −0.027
GD → PWS → PA avoidance → PA	−0.003 (0.003)	−0.004	−0.010, 0.002
GD → PWS → IWS → PA avoidance → PA	−0.017 (0.005)	**−0.017**	−0.028, −0.008

GD = gaming disorder; PA = physical activity; IWS = internalized weight stigma; PWS = perceived weight stigma; Est. (S.E.) = estimate coefficient (standard error); Std. coeff. = standardized coefficient; CI = confidence interval. Significant findings are shown in **bold**.

## Discussion

The present study was conducted to investigate the direct association between gaming disorder (GD) and physical activity (PA), and how intermediate variables including perceived weight stigma, internalized weight stigma, and PA avoidance may indirectly mediate this association. The findings were supportive of H_1_ indicating the significant pathways between GD and PA, and also found that GD was significantly associated with intermediate variables (i.e., PA avoidance and stigma measures). However, only PA avoidance and perceived weight stigma were associated with PA. In addition, three mediation effects, namely (i) solely PA avoidance, (ii) internalized weight stigma followed by PA avoidance, and (iii) perceived weight stigma, internalized weight stigma then PA avoidance, negatively mediated the association between GD and PA. These findings supported H_2_, H_5_ and H_7_, but not H_3_, H_4_ or H_6_. Therefore, higher GD was associated with higher PA, but may be associated with lower PA via the negative mediation effect of PA avoidance and weight stigma.

Of initial note was that GD showed a positively direct association with PA, but the number of individuals with gaming-related negative consequences and cognitive behavioral symptoms was relatively higher among individuals with lower levels of PA. A consistent finding was found in a previous study conducted by Huang et al. (2022) which assessed the temporal associations between PA and forms of problematic use of the internet including gaming. Both the present study and the one conducted by Huang et al. (2022) found a positive association between problematic gaming and PA level. However, several studies support the hypothesis that any problematic gaming behavior, particularly some specific types of GD (e.g., IGD), may relate inversely to PA. Cheng et al. (2022) and Kwok et al. (2021) found that PA was negatively associated with internet addiction/IGD among college students, and Pirnes et al. (2022) reported a negative association between PA and screen playing times among school-aged adolescents. These contradictory findings suggest a complex relationship between PA and problematic use of the internet including online gaming, suggesting that other factors, unmodelled here, likely account for the positive correlation between GD and PA observed directly (although these were not identified in the present study).

Another novel finding in the present study was the mediating effect of PA avoidance in the association between GD and PA. The results indicated that individuals with an elevated risk of GD may demonstrate higher levels of PA avoidance, and PA avoidance may negatively relate to PA, mediating an inverse relationship between GD and PA in the opposite direction of the direct effect, contrary to traditional mediation effects. One potential explanation for such findings may involve individuals affected by GD experiencing hyperactivity that may increase their engagement in PA. However, because escapism and avoidance coping have been reported as a predictor of GD (Melodia, Canale, & Griffiths, 2020), co-existing characteristics of GD (e.g., low self-esteem) may also contribute to the likelihood of developing PA avoidance (Moral-García, García, García, Jiménez, & Dios, 2018), which has also been associated with reduced PA (Yi et al., 2024). As these possibilities are speculative, further investigation is warranted to clarify not only direct associations between GD and PA, especially when factors such as types of gaming, duration, and severity of the problem may play determinative roles in such associations, but also how potential intermediate factors may operate in such relationships. Other underlying and potentially confounding factors should also be explored.

In the present model, which included a population with average BMIs, weight stigma did not appear strongly related to PA. Previous studies have reported mixed findings regarding the association between weight stigma and PA level (Bevan et al., 2021, 2022; Pearl et al., 2021). Among individuals who are overweight or have obesity, both perceived weight stigma and internalized weight stigma were supported as contributors to PA avoidance and may result in low PA (Han, Agostini, Brewis, & Wutich, 2018). When studying populations with average BMIs, internalized weight stigma has more frequently been reported to associate with low PA (Huang et al., 2024; Pearl et al., 2015, 2021).

However, in the present model, which comprised participants with average BMIs, GD acted as a stronger factor that overtook the influence of weight stigma on PA, independently or via the impact of PA avoidance which (likely) subsequently affected PA levels. Therefore, individuals with higher levels of gaming-related negative consequences and cognitive behavioral symptoms may have higher PA avoidance and show less motivation for PA engagement than others, resulting in less PA. Although the direct and indirect effects of GD on PA avoidance should be investigated through experimental and longitudinal studies, such associations may consider GD as a potential risk factor for impaired motivation toward PA in some (but not all) individuals, and should be investigated in future studies. It was also found that compared to males, females reported higher levels of PA avoidance and weight-based stigma. This finding has been reported in previous studies (Ajibewa et al., 2022; Bevan et al., 2021, 2022) and may be related to different understandings or perceptions between females and males regarding these concepts and the amount of value or importance that females and males place on these factors.

The demographic variables (e.g., age, BMI, and gender) assessed in the present study implicated age, with those of younger age being more likely to have greater PA. Individuals with high levels of PA were also more likely to have lower GD severity and PA avoidance. Adolescence/emerging adulthood may be accompanied by decreased participation in PA and sports because work and family responsibilities may change (Harridge et al., 2017). In addition, a study by Greenleaf et al. (2020) examining the associations between body image and exercise avoidance found that those who reported greater motivation to exercise experienced less exercise avoidance, consistent with the finding in the present study that found an association between high PA and low PA avoidance.

However, there are limited data regarding whether PA may have a protective effect against GD. Henchoz et al. (2016) conducted a longitudinal study among emerging adults and identified reciprocal relationships between level of PA/sports and GD. The results suggested that individuals with lower levels of PA and more sedentary behavior may be more susceptible towards developing GD, and GD may preclude PA. However, improved PA engagement may prevent GD development, and even reverse lower PA engagement among gamers with GD, suggesting the importance of PA promotion in preventing GD consequences. Despite the present finding suggesting positive associations between GD and PA, other negative influences of GD (e.g., withdrawal behaviors or mental health problems due to GD) should not be ignored. Consequently, healthcare providers still need to consider how to help individuals actively improve their PA.

The present study used a complex approach to investigate the associations between the GD and PA through both mediation and moderation analyses. In the simple model (model a), the significant negative mediating roles of PA avoidance and perceived weight stigma were confirmed, and there was a direct significant effect between the GD and PA. However, in three serial mediation models (models b, c, and d), positive associations of both types of weight stigma with PA avoidance, along with negative association of PA avoidance with PA, were found, but the associations between GD and PA became inconsistent. This means that if all the mediators are placed in parallel positions, GD would be positively correlated with PA, but this would only happen when the actual impacts of weight stigma on PA avoidance were neglected. Therefore, considering the literature supports the role of both types of weight stigma in the development of PA avoidance (Bevan et al., 2021, 2022; Huang et al., 2024), it is better to put PA avoidance after both types of weight stigma, to study the effect of weight stigma in contributing in PA avoidance. In such situations, the direct association between GD and PA was not present, indicating that the sequential order of mediators would be more effective in explaining the underlying mechanism of how GD may negatively affect PA.

Moreover, because the literature supports the fact that internalized weight stigma may occur when individuals have previously experienced perceived weight stigma (Durso et al., 2012; Han et al., 2018), placing perceived weight stigma at a position before internalized weight stigma would additionally provide a clear chain of mediators that may play any role in defining the indirect associations between GD and PA. Therefore, as the results of serial mediation indicated, GD through its positive association with perceived weight stigma, followed by positive association with internalized weight stigma, may contribute to the development of PA avoidance, which may ultimately negatively impact PA behavior.

The present study has some limitations. First, the cross-sectional design precludes causal inferences. Second, an online platform was used to distribute the survey and collect data, which may be vulnerable to biases (e.g., selection bias). Third, the participants only included individuals aged between 20 and 40 years old despite the fact that both GD and lower PA are prevalent among younger populations (i.e., adolescents). Young adults were particularly targeted considering they have a higher degree of freedom than students or parents in their daily routine (e.g., live independently, less family responsibility). However, assessing the present study's hypotheses among a younger population should be considered in future studies. Fourth, data regarding sample's gaming habits, such as types of activities, devices used, online/offline games, were not collected. This information may be practical for understanding details of context that may contribute in GD, and its associations with other study variables. Fifth, given that studies indicate male gender appears to be a risk factor for GD (Kim et al., 2022), less than 40% of the present study's participants were males. This may affect the generalizability of the findings for both genders. Finally, a general sample was used and this may have had different characteristics relative to individuals with clinical concerns. For example, the level of PA avoidance or weight-based stigmatization may differ among individuals who are overweight or have obesity compared to population with average weight. Therefore, studying individuals diagnosed with GD or states directly related to weight stigma are indicated. Based on the present findings, in addition to strategies in reducing GD, interventions such as providing stigma-free environments or motivational interviewing (Huang et al., 2024) may potentially be adopted to reduce PA avoidance and public weight stigma. Additional strategies should be adopted to foster the engagement of PA because it may reduce PA avoidance, which may promote engagement in PA, especially among individuals with elevated risk for GD.

## Conclusions

The present study showed that intermediate variables (i.e., tendency for PA avoidance and perceived weight stigma) had significant associations with both GD and PA among Taiwanese young adults. Moreover, these intermediate variables mediated the relationship between GD and PA oppositely from the direct relationship. That is, higher GD was associated with higher PA, but associated with lower PA through the mediation of PA avoidance and both types of weight stigma. Strategies to reduce GD or provide stigma-free environments are recommended in order to improve PA engagement. Additional approaches for fostering engagement in PA are also suggested to reduce PA avoidance and its subsequent impact on PA. Investigations of clinical samples (e.g., individuals with a confirmed diagnosis of GD or who are overweight or have obesity) are needed to understand mechanisms among patients.

## Appendix

**Table A1.** Summarized moderated effects of weight stigma and physical activity avoidance in the relationship between gaming disorder and physical activity

Independent variable	B	SE	*t*	*p*-value	LLCI	ULCI
GADIS-A	185.875	184.722	1.006	0.315	−176.674	548.423
TAPAS	−43.243	14.679	−2.946	0.003	−72.053	−14.434
GADIS-A* TAPAS	−2.344	5.256	−0.446	0.656	−12.659	7.971
GADIS-A	261.103	238.039	1.097	0.273	−206.091	728.297
WBIS	32.139	18.087	1.777	0.076	−3.359	67.638
GADIS-A* WBIS	−4.179	6.342	−0.659	0.510	−16.626	8.268
GADIS-A	83.862	79.713	1.052	0.293	−72.589	240.313
PWSS	−85.494	56.882	−1.503	0.133	−197.135	26.146
GADIS-A* PWSS	8.582	18.282	0.469	0.639	−27.301	44.464

B = unstandardized coefficient; SE = standard error; LLCI = lower limit confidence interval; ULCI = upper limit confidence interval; GADIS-A = Gaming Disorder Scale for Adolescents; TAPAS = Tendency to Avoid Physical Activity Scale; WBIS = Weight Bias Internalization Scale; PWSS = Perceived Weight Stigma Scale.

International Physical Activity Questionnaire (IPAQ) in total scores was set as dependent variable. GADIS-A*TAPAS is the moderated effect of physical activity avoidance; GADIS-A*WBIS is the moderated effect of internalized weight stigma; GADIS-A*PWSS is the moderated effect of perceived weight stigma.
